# Mining and analysis of public sentiment during disaster events: The extreme rainstorm disaster in megacities of China in 2021

**DOI:** 10.1016/j.heliyon.2023.e18272

**Published:** 2023-07-14

**Authors:** Zheng Qu, Juanle Wang, Min Zhang

**Affiliations:** a School of Civil and Architectural Engineering, Shandong University of Technology, Zibo 255049, Shandong, China; b State Key Laboratory of Resources and Environmental Information System, Institute of Geographic Science and Natural Resources Research, Chinese Academy of Sciences, Beijing 100101, China; c University of Chinese Academy of Sciences, Beijing 100049, China; d Jiangsu Province Geographic Information Resources Development and Utilization Collaborative Innovation Center, Nanjing 210023, China

**Keywords:** Public sentiment, Social media, Mega rainstorm disaster, Flood hazard, Urban disaster reduction

## Abstract

Cities are concentrated areas of population that are vulnerable to the impact of natural disasters. Owing to the impact of climate change and extreme weather incidents in recent years, many cities worldwide have been affected by sudden disasters, especially floods, causing many casualties. Social media plays an important role in the communication and sharing of information when physical communication is limited in emergency situations. However, obtaining and using public sentiment during major disasters to provide support for emergency disaster relief is a popular research topic. In the summer of 2021, China's inland plains experienced extremely serious rainstorms. The rainfall on July 20 in the capital city of Zhengzhou, Henan Province, the most population province in China, reached 201.9 mm/h, causing extremely serious consequences. This case study examines people's sentiment about this event through datamining of Chinese Weibo social media during the extreme rainfall period. The six most concerned types of public response topics and 14 subcategory topics were determined from 2,124,162 Weibo messages. “Asking for help” and “public sentiment” dominated the main topics, reaching almost 66%, with a relatively even distribution of secondary categories, but with “appeal for assistance” taking the top spot. Topics changed cyclically with work and rest, but these areas seemed to lag behind coastal areas in their responses to the storm in the same time. The topics were centred around Zhengzhou and distributed in China's major city clusters, such as the Beijing-Tianjin-Hebei agglomerations, Yangtze River Delta, and Pearl River Delta regions. Community-level disaster relief information was also discovered, which showed that high building power outages, basement flooding, tunnel trapping, and drinking water shortages were common topics in specific inner urban regions. This detailed information will contribute to accurate location-based relief in the future. Based on this lesson, a series of measures for urban flood reduction are proposed, including disaster prevention awareness, infrastructure building, regulation mechanisms, social inclusivity, and media dissemination.

## 1 Introduction

Cities are the main areas where life and work are concentrated. According to statistics from the United Nations, the world's urban population increased from 751 million in 1950 to 4.2 billion in 2018. The proportion of the urban population has increased from 30% to 55%, and is expected to increase to 68% by 2050 [1]. Urban floods caused by heavy rain pose a major threat to the safety of people and property [2]. Sudden flood disasters can easily lead to casualties and property loss in urban areas. In 2021, the economic losses caused by global floods reached 82 billion US dollars, accounting for 31% of global natural disaster-related economic losses [3]. During the 2022 York City floods in the United Kingdom, 12 h of total downshift of 58 mm caused 400 real estate units to be flooded [4]. In the 2021 Birmingham City floods in the United States, the rainfall per hour reached 100–130 mm, causing four deaths and damaging 250 households [5]. In Asia-Oceania regions, the 2022 Sukkur City floods in Pakistan, with an average annual precipitation of 388.7 mm, marked an increase of 190% over the previous 30 years, causing 1300 casualties in the south and a loss of $ 40 billion [6]. During the 2022 Brisbane City floods in Australia, the maximum rainfall exceeded 400 mm, which caused 22 casualties and a loss of A$ 2 billion [7]. The precipitation in Hiroshima County and Okayama Prefectures in Japan in 2018 exceeded 300 mm of daily rainfall, causing 225 casualties and losses of 8 billion US dollars [8]. In 2021, in China's most populous province, the largest hour of rainfall experienced by Zhengzhou, Henan Province, reached 201.9 mm, causing 380 deaths and missing persons and 40.9 billion yuan losses [9]. In the face of such an experience, strengthening responses to floods is of great significance.

During a flood, owing to the disaster conditions, the public lacks convenient physical connections, and social media has become the main channel for information release and dissemination because the Internet allows everyone to participate in media communication as a producer or consumer of disaster response information. The public's response to various disaster topics and the popularity of discussions also reflects the masses' awareness of disaster events. The large amount of geographical information and disaster events on social media has become an important source of data for flood disaster risk reduction.

Many scholars have used social media to analyse the evolution of disaster events. Sakaki et al. [10] collected Twitter data of earthquakes in Japan, and proposed a support vector machine (SVM) algorithm to mine text, speeding up the dissemination of disaster events in social media. Liang et al. [11] collected data from 27,218 tweets during Typhoon Meranti using sign-in location information, to demonstrate the spatiotemporal process trend of the disaster. Zhang et al. [12] analysed 25,798 microblogging data during the typhoon Mangkhut disaster in 21 prefecture-level cities, and found the negative impact of the disaster event on public sentiment. Li et al. [13] gathered 288,184 microblogging data and analysed the evolution of public opinion for two different typhoons in Guangdong Province, and found the relationship between people's sentiment and economic losses. Han et al. [14] analysed 28,608 microblogs related to the floods in Shouguang, divided into six related topics, and found that condemning the government has the highest voices. Liu et al. [15] studied 26,059 “7.21 Beijing rainstorm” microblogs and divided them into seven disaster-related information themes to obtain findings on the impact of rainstorms on road traffic through the distribution of hotspots. Alomari et al. [16] collected 190,000 tweets in response to COVID-19, based on a Saudi Arabian scope and divided into six macro themes, and found that blood donation and treatment in hospitals was a major concern during the pandemic. Yigitcanlar et al. [17] collected 131,673 tweets in Australia, and found that the negative emotions of the public can be used to find what is damaged and need help.

Previous studies have mostly analysed disaster public opinion from the perspective of social impact and government decision-making, but have lacked links between the large spatial scale and the local community scale, which can lead to a disconnect between the analysis of disaster public opinion and emergency response capacity. This study considers the Zhengzhou rainstorm, the most severe rainstorm event in China in recent years, as the subject, and conducts a national-scale analysis of public response during a rainstorm disaster as well as a local-scale analysis of disaster information at the city and community levels, proposing targeted urban rainstorm disaster response recommendations to support the prevention and control of flooding in megacities.

## 2 Materials and methods

### 2.1 Research data

**Fig. 1:**
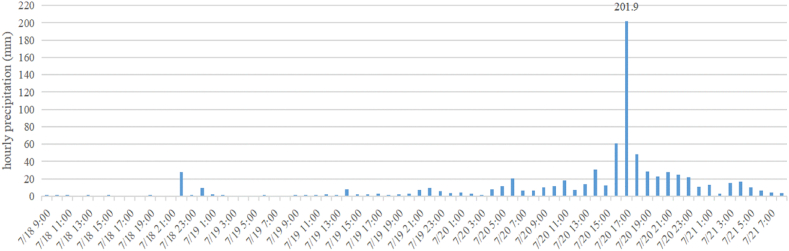
Zhengzhou National Meteorological Station 18 July 08:00–21 July 08:00 Hourly precipitation change.

From July 17 to 23, Henan Province experienced a historically rare heavy rainstorm. From the 19th to the 20th, the centre of the rainstorm moved south to Zhengzhou, and a long and long-lasting heavy rainstorm occurred, such that from 16:00 to 17:00 on the 20th, the rainfall at Zhengzhou Station reached 201.9 mm (Fig. 1), reaching the historical extreme value of Chinese mainland rainfall. Rare and sustained heavy rains have caused severe urban waterlogging in Zhengzhou.

Social media data were collected from Weibo data in Chinese (Chinese Twitter), with 5s as the minimum search unit and “Zhengzhou” as the keyword, from 0:00 on July 17 to 0:00 on July 28, 2021. The selected time span covered the rainfall stage before the disaster, relief stage, and recovery period after the disaster. The microblog message information includes attribute fields, such as username, user ID, microblog content text, geographic location, and publishing time. This study used keyword searching to remove text containing irrelevant terms. After removing repeated microblog texts, 2,124,162 pieces of microblog data remained. The Chinese text segmentation Python package (Jieba) was used for the microblog data segmentation. Combined with relevant domain knowledge, we supplement the characteristic vocabulary of rainstorm types, such as “extraordinary rainstorm”, “thunderstorm”, “storm”, “heavy rain”, “moderate rain”, and “rainstorm red warning”, to create a word segmentation dictionary suitable for rainstorm disasters. We removed punctuation marks, prepositions, advertisements, and other irrelevant words to effectively process the data.

Auxiliary data included DEM (Digital Elevation Model), land use classification, and word cloud data from Weibo topic segmentation. The DEM data for Zhengzhou were derived from the Geospatial Data Cloud of the Chinese Academy of Sciences. ASTER GDEM 30 M resolution digital elevation data were used as the data source. Land-use classification data were derived from the Sentinel-2 satellite remote sensing map with a spatial resolution of 10 m in 2021, produced by the Environmental Systems Research Institute, Inc. (ESRI) in the US. Word cloud data of Weibo topic segmentation were created with 300,000 words as the basic data, and software for translating word cloud data was provided by the Institute of Science and Technology Information of the China Academy of Railway Sciences Group Co., Ltd.

### 2.2 Data pre-processing and methods

The LDA (Latent Dirichlet Allocation) topic-mining method was used to construct the storm disaster information-mining model. It is a Bayesian probability model proposed by Beli et al. [18]. With three layers of ‘document-topic-word’, that forms topics through clustering from information in massive texts, where documents are represented as a random mixture over latent topics, each of which is characterised by a distribution of words. The LDA model is implemented using the “Gensim” package in Python. For repeated experiments using the LDA model, the optimal number of initial topics was set to 10. Based on the topic glossary, and after merging similar topics and eliminating irrelevant topics, the final summary contained six primary topics.

Using the seasonal trend decomposition method based on LOESS (Loess, STL), the overall time trend of microblog topics related to rainstorms in Zhengzhou was analysed [14]. The STL algorithm uses LOESS to smooth the time series in two loops: the inner loop iterates between seasonal and trend smoothing, and the outer loop minimises the effect of outliers. During the inner loop, the seasonal component is first computed and then removed to compute the trend component, which is computed by subtracting the seasonal and trend components from the time series. Using SPSS software, STL was used to extract seasonal rainstorm-related microblogs from the quantitative time series. As shown in Equation (1), the time series can be considered as the sum of three components: a trend component, seasonal component, and a remainder in STL.

(1) X_t_ = T_t_ + S_t_ + R_t_

Where *X*_*t*_ is the original time series, *T*_*t*_ is the trend component, *S*_*t*_ is the seasonal component, and *R*_*t*_ is the residual component.

Kernel density analysis was used to examine the spatial distribution characteristics of microblog topics related to rainstorms in Zhengzhou. The primary purpose of the Kernel Density tool is to calculate the density of point features around each output raster cell. Conceptually, we first define a neighbourhood around the centre of each raster pixel, then add the number of points in the neighbourhood, and divide by the total neighbourhood area to obtain the density of the point features. The principle is to use Equation (2) to determine the search radius (also known as the bandwidth), and by using the quadratic kernel function to generate a smooth surface to detect the intensity of the event. Let (s_1_, …, s_i_, …, s_n_) be a series of event samples distributed with an unknown density, $\hat{\lambda }\left(s\right)$, in the study area. Its kernel density estimator is Equation (3).

(2) $$SearchRadius=0.9*\min\left(SD,\sqrt{\frac{1}{\ln\left(2\right)}}*D_{m}\right)*n^{-0.2}$$

where *SD* is the standard distance and *D*_*m*_ is the median distance. The min part of the equation indicates that the smaller of the two options (SD or $\sqrt{\frac{1}{\ln\left(2\right)}}*D_{m}$) will be used to calculate the value.

(3) $$\hat{\lambda }\left(s\right)=\sum_{i=1}^{n}\frac{1}{\tau^{2}}k\left(\frac{s-s_{i}}{\tau }\right)$$

where k is the kernel function, τ is a smoothing parameter called the bandwidth, i.e., the search radius within which to calculate density, and s-s_i_ is the distance between s and s_i_.

## 3 Results

### 3.1 Disaster evolution trend

**Fig. 2:**
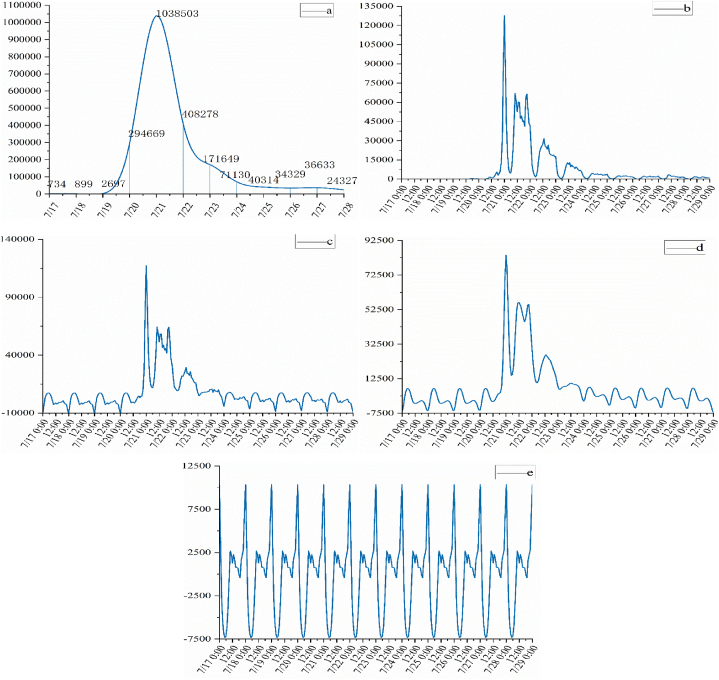
Time trend seasonal decomposition.

Relevant microblog text data for rainstorms are shown in Fig. 2. Fig. 2(a) shows the original time series of the number of rainstorm-related microblogs divided by day. Before July 20, when it was not determined that the rainfall in Zhengzhou would develop into a heavy rainstorm, there was almost no online user discussion on the topic of “Rainstorm in Zhengzhou”. On the 20th, heavy rainstorms struck Zhengzhou City, and with the outbreak of urban flooding, public opinion rose sharply. A total of 1,038,503 Weibo data items were crawled on July 21 the peak of the statistics, with an increase of about 252.4% compared to the 20th. At 13:00 on July 22, the Zhengzhou Flood Control Headquarters decided to reduce the flood control Level I emergency response to Level III and the data began to decline gradually. It shows that the trends of urban flooding extracted from the tweets are consistent with the actual disaster phases. Fig. 2(b) shows the original time series of the number of rainstorm-related microblogs divided by hour. The number of related microblogs increased slightly at 16:00 on July 20, reaching its first small peak at 19:00, and subsequently increased substantially. The Zhengzhou Meteorological Observatory issued a red rainstorm warning at 0:25 on July 21. The Weibo data reached its peak at 0:00 on July 21. The number of Weibo data items collected was 127,581 in just 1 h, and the cyclic fluctuations declined thereafter. Because the original time series did not have a long-term trend (over a long period, the trend of continuous increase or decrease), it was judged to have seasonal changes, and the method of seasonal prediction in the SPSS software was used to decompose the original series into seasonal, trend, and residual components. Fig. 2(c) is a seasonally adjusted time series showing the trend in the number of flood-related microblogs after removing seasonal factors. Fig. 2(d) shows the part where the number of flood-related microblogs changed periodically. With the development of events and the influence of people's work and rest, the daily volume of microblogs sent has two peaks. The morning peak usually occurs around 10:00, the evening peak time is usually around 23:00, and the trough period is between 0:00 and 6:00 every day. Fig. 2(e) shows the trend component, which reflects the trend in the number of disaster-related microblogs.

### 3.2 Topic classification results

After repeated testing, six first-level microblog topics were identified (Table 1). As LDA is an unsupervised topic model, it must be summarised manually based on keyword information, such as the classification of information containing keywords such as “rainfall” as “Weather Conditions”, “help” as “Rescue Information”, “public welfare” as “Assistance Information”, “sad” as “Public Sentiment”, “people's daily” as “Official Notification”, and “section” as “Traffic Conditions”. Statistics on the number and proportion of their microblogs (Table 1). Disaster events grab a great deal of public concern and many people use social media to express their emotions. In this flood event, the damage caused was severe and nearly 1/3 of the topics were disaster relief requirements. This also reflects public response in social media has rich information for rapid emergency response.

**Table 1:** Classification statistics of different topics on Weibo.

Topic	Number of Weibo	Proportions
Public Sentiment	747,679	36.9%
Asking for Help	581,201	28.7%
Assistance Information	329,091	16.2%
Weather Forecast	185,808	9.2%
Official Notice	139,882	6.9%
Traffic Conditions	43,853	2.2%

**Fig. 3:**
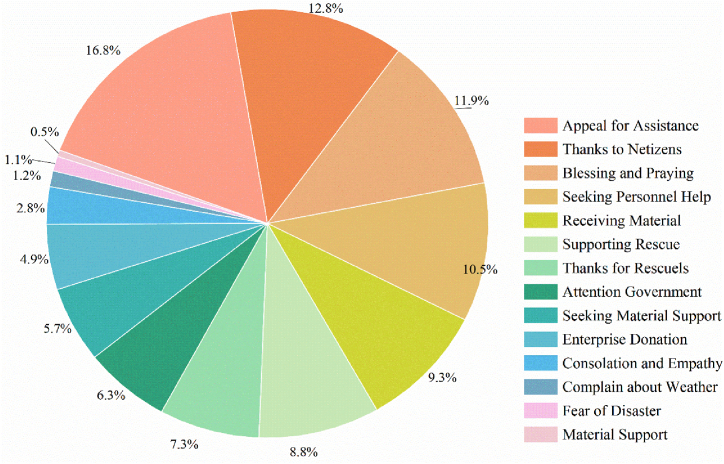
Secondary topic statistics.

A secondary classification of the three primary topics of “Assistance Information”, “Asking for Help”, and “Public Sentiment” was then performed. Fig. 3 presents the statistical results of the number of microblogs in the 14 topic categories at the second level. “Appeal for Assistance”, “Thanks to Netizens”, “Blessing and Praying”, and “Seeking Personnel Help” were the four most common public sentiments during the heavy rain in Zhengzhou, with Weibo posts accounting for 16.7%, 12.7%, 11.8%, and 10.4%, respectively, for a total of 51.6% of all posts. In terms of mass emotions, positive emotions such as gratitude for the attention of netizens (12.7%), thanks for rescue (7.3%) and prayers for those affected by the disaster (11.8%) are higher than negative emotions such as fear of natural disasters (1.1%) and complaints about the weather (1.2%). This shows public have confidence for inland flooding, while also implies people easily underestimate the potential damages of extreme rainfall. The details of the rescues further distinguish between the need for relief people (10.4%) and materials (5.7%).

#### 3.2.1 Topic temporal trend

To accurately display the temporal changes in different topics, the number of Weibo texts for each topic and emotion was counted at 12-h intervals. The statistical results of secondary specific classifications are illustrated in Fig. 4a, Fig. 4ba, 4b. The first pole was reached for all topics at 00:00 on July 21. As the rainstorm continued, the topic of “Consolation and Empathy” was always active, peaking many times, and there were also obvious fluctuations from the 22nd to the 23rd. The topic of “Complain about Weather” reached its second extreme in the middle of the rainstorm when urban floods were formed, above 0:00 on the 21st, and still be concerned until 27th. By 16:00 on the 22nd, the Zhengzhou Red Cross Society had received a total of 497 million yuan (at the society's own level) in donations and 698,100 yuan in materials. At 19:37 on the 21st, Sina Henan announced that Zhengzhou had 41 temporary water-intake points. “Supporting Rescue”, “Thanks for Rescue”, “Blessing and Praying”, “Attention Government”, and “Receiving Materials”second peak appeared at approximately 20:00 on the 21st. On July 21, many companies donated money to help Henan, and “Enterprise Donation” attracted attention frequently, reaching the second peak at around 13:00 on the 21st. “Material Support”, “Seeking Material Help”, and “Appeal for Assistance” displayed an inverted pyramid fluctuation, and received significant attention during the flood period on the 22nd. The various topics show that as the torrential rains continued, public have more complaints initially, more thanks emerged as the rescue effort progressed, on the 22nd. As the city is trapped over an extended period of time, the urgent need for material help gradually appeared in the later stages of the disaster.

**Fig. 4a:**
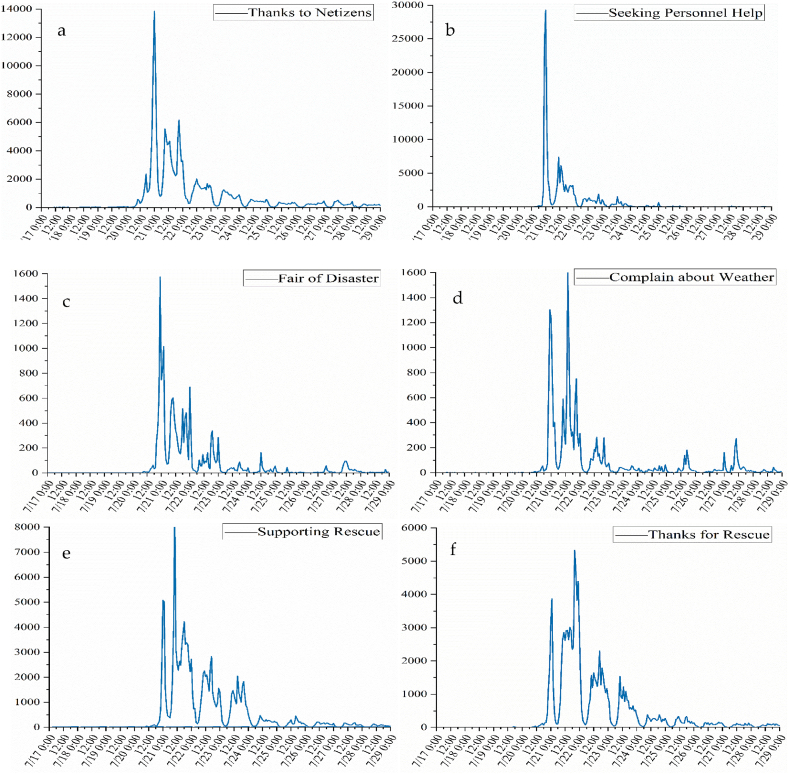
Secondary topic temporal series (a: Thanks to Netizens, b: Seeking Personnel Help, c: Fair of Disaster, d: Complain about Weather, e: Supporting Rescue, f: Thanks for Rescue, g: Blessing and Praying, h: Attention Government, i: Receiving Materials, j: Enterprise Donation).

**Fig. 4b:**
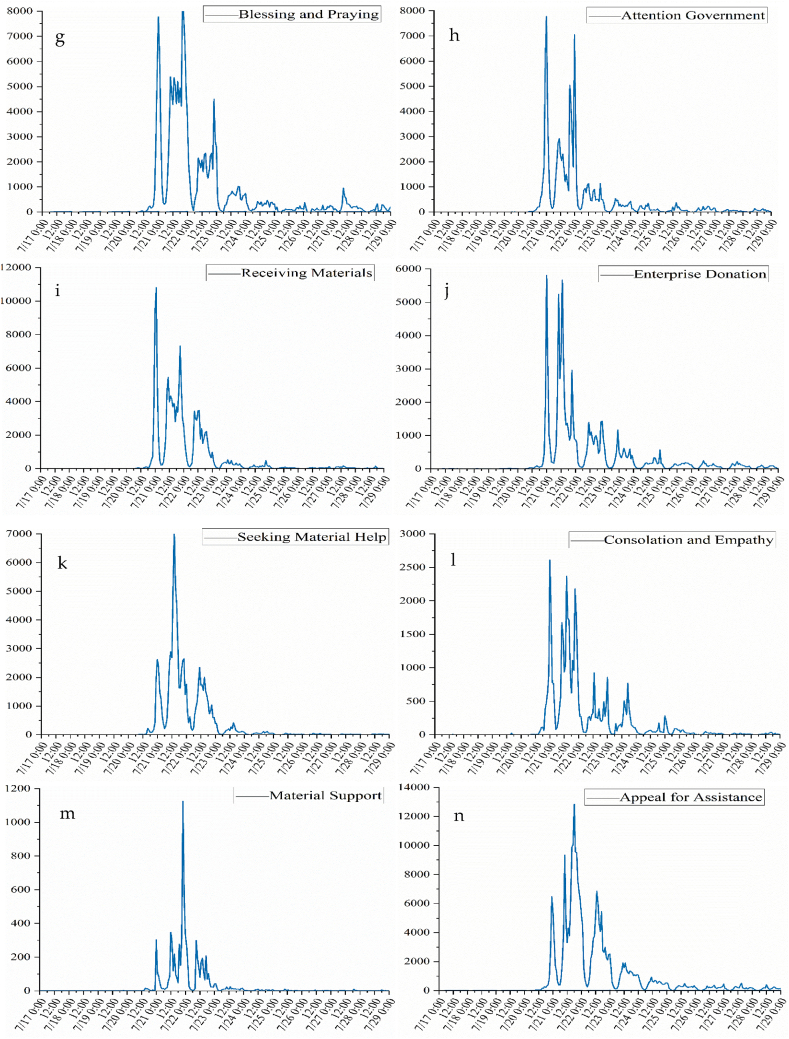
Secondary topic temporal series (k: Seeking Material Support, l: Consolation and Empathy, m: Material Support, n: Appeal for Assistance).

**Fig. 5:**
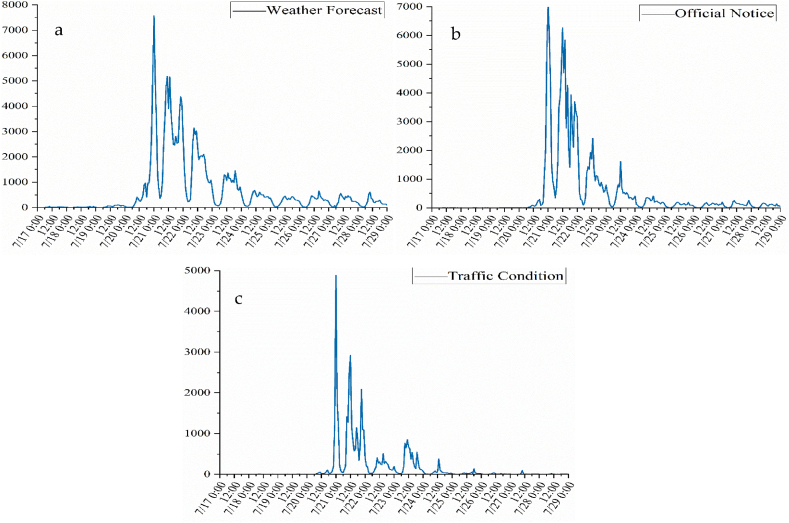
First-level topic temporal series (a: Weather Forecast, b: Official Notice, c: Traffic Condition).

The first-level thematic time series without secondary classification is illustrated in Fig. 5. The overall trend was more consistent across the three topics, the curve began to grow rapidly at 22:00 on the 20th, peaked at 00:00 on the 21st, and then fluctuations drop. Of these, the “Weather Forecast” have strong concerning by people in the whole disaster process. “Official Notice” and “Weather Forecast” were almost simultaneous in the disaster period, reflecting the fact that the government's voice was known as soon as the disaster struck. “Traffic Condition” was synchronised with the intensity of the flooding and were highest on the 21st, probably because of the main transportation system broken brought more concerns, especially the subway submerged.

#### 3.2.2 Topic spatial distribution

The overall spatial distribution of topics was concentrated in Henan Province and extended outwards; however, the direction of expansion varied according to the mood of people in different regions [19]. This study focused on the spatial distribution of secondary topics in Weibo sentiments (Fig. 6a, Fig. 6ba, 6b), which exhibited the characteristics of more to the east and less to the west, and heavier to the south and lighter to the north. “Attention Government”, “Enterprise Donation”, “Blessing and Praying”, “Supporting Rescue”, mainly concentrated in the Henan Province and population clusters such as Beijing-Tianjin-Hebei, Sichuan-Chongqing urban agglomeration, Yangtze River Delta, Pearl River Delta, etc. “Thanks to Netizens” and material related topics were more concentrated in Henan Province, where the disaster occurred. In addition, emotional topics such as “Fear of Disaster”, and “Complain about Weather” were also discussed in Shanghai, Nanjing, Guangzhou. These analyzes show that people living in areas prone to rainstorm disasters (e.g. coastal regions) had more sensitive for this kind of disasters and paid more attention to this event. More information on specific disaster relief needs is concentrated in the disaster-affected areas.

**Fig. 6a:**
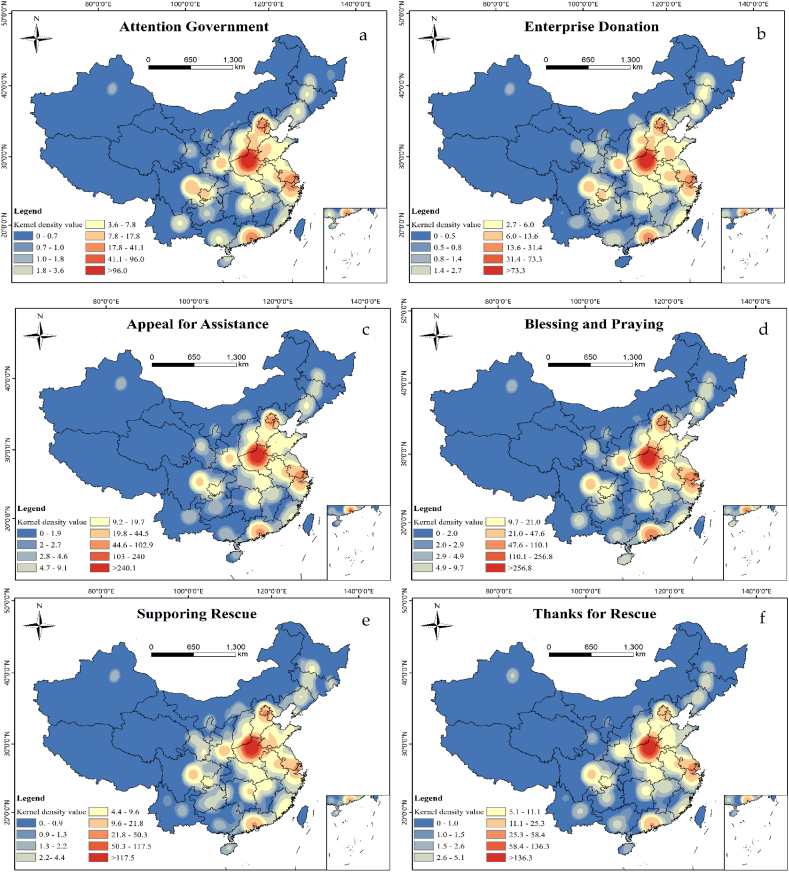
Spatial distribution of Weibo topics (a: Attention Government, b: Enterprise Donation).

**Fig. 6b:**
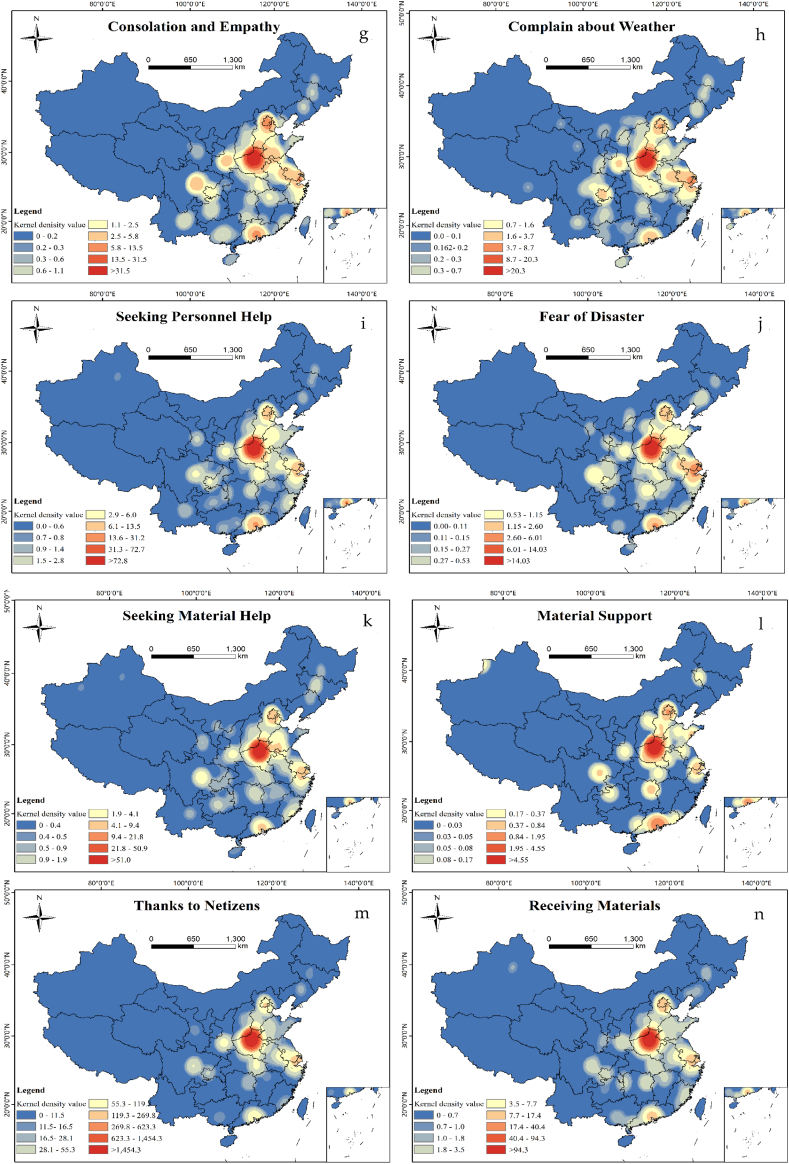
Spatial distribution of Weibo topics (c: Appeal for Assistance, d: Blessing and Praying, e: Supporting Rescue, f: Thanks for Rescue, g: Consolation and Empathy, h: Complain about Weather, i: Seeking Personnel Help, j: Fair of Disaster, k: Seeking Material Support, l: Material Support, m: Thanks to Netizens, n: Receiving Materials) (search radius = 200 km).

### 3.3 Community topic analysis

**Fig. 7:**
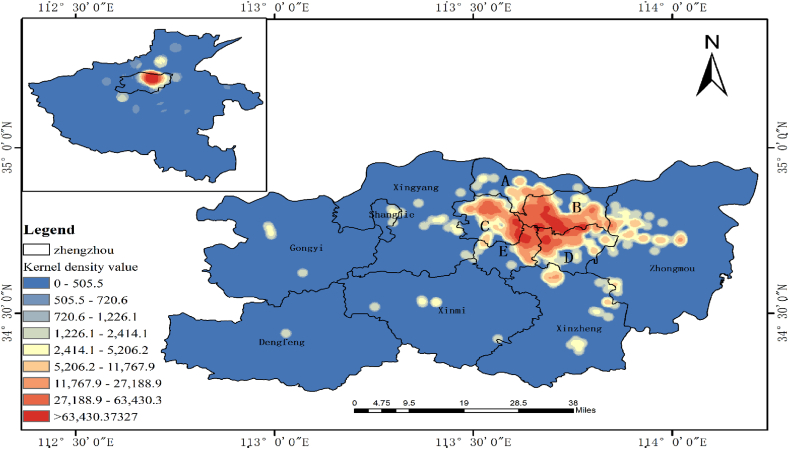
Spatial distribution of Weibo topics in Zhengzhou city (search radius 2 km).

To further explore disaster information on Sina Weibo, the community in Zhengzhou was used as a research unit to extract public opinion. Approximately 42,111 pieces of residential community-related text data were extracted from the obtained texts, and 2217 text data with geographical locations were screened. Spatial kernel density analysis was conducted using the text, and the spatial distribution map is shown in Fig. 7. From the perspective of spatial distribution, the topic mainly focused on Zhengzhou City, which verifies that the topic originated from the interior of the disaster site. Taking Zhengzhou City as the research object, narrowing the kernel density search radius, and analysing the kernel density value, it is clear that the topics are mainly distributed in the inner urban area, including A: Huiji District, B: Jinshui District, C: Zhongyuan District, D: Guancheng Hui Nationality District, E: Erqi District, and the central part of Zhongmu County near the urban area, with a large core and multiple small cores. The central urban area covers only 16.44% of the area of Zhengzhou, but accounts for 54.26% of the population. The central urban area has a well-developed transportation network, urban infrastructure, dense residential areas, commercial areas, and schools [20]. It is further verified that the spatial distribution of topics in the Weibo texts is correlated with the economic level and degree of urbanisation.

**Fig. 8:**
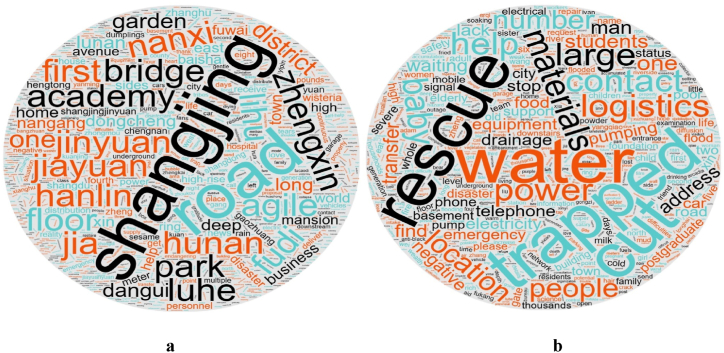
a Communities affected by the disaster. b Specific needs of the affected areas.

Text screening was carried out with “hard-hit areas” as the secondary keyword, and word segmentation extraction was visualised. The word cloud shown visualises the frequency of individual words in the data corpus, where larger words were mentioned more frequently. It is clear that the names of the main extracted areas are highly correlated with the kernel density distribution, as shown in Fig. 8(a). Shangjing, Jinyuan, Shangjingjiayuan, Longhu Park, Zhongjian Han Forest Garden, and Hunan Road were located in Area B, and Dongcheng Garden was located in Area D. Nanxiyuan was located in Area E, Agile, Central Garden, Zhengxin Park, Lihuqiao, Fuwai Hospital, Zhengxin Road, and other places in Zhongmou County. These word cloud messages are mainly located near main urban areas, which is a side effect of the positive responses of relatively densely populated and economically advanced communities to disaster public opinion messages through social media.

“Urgent need” is used as the secondary keyword for topic screening and word cloud display (Fig. 8(b)). Material transport is extremely important, and the main material resources are drinking water, food, and large water pumps. Urban infrastructure issues requiring urgent improvement include logistics failure, power outages, Internet disconnection, lack of mobile signals, and basement submergence. Disaster relief issues that can easily be overlooked are vulnerable groups such as missing people, the seriously injured, the elderly, infants in urgent need of help, and high-building households that experience difficulty receiving relief resources. By visualising the word cloud of community text information, more specific information about severely affected communities can be extracted, which can contribute to accurate and timely location-based disaster relief.

## 4 Discussion

### 4.1 Topic temporal series analysis

The results of the mining and analysis of public opinion on disasters are closely related to the real-time development process of disasters. Based on an analysis of the Zhengzhou rainstorm warning and public response, the period when the public response was hot lagged slightly behind the rainstorm warning period. The peak of the overall topic data of the Zhengzhou rainstorm microblog appeared on the 21st, that is, in the middle of the rainstorm, when the amount of precipitation had started a downward trend but there was still heavy rain. In fact, the Zhengzhou weather station issued a “yellow rainstorm warning’ on the 18th and an “orange rainstorm warning” on the 19th. Second, the masses have a fixed amount of time to access the internet in the morning and evening, and this time characteristic is more consistent with the research results of other scholars [14]. Third, compared with the public opinion research data of other disasters, there was a notable lag in the time node of the Weibo peak of the Zhengzhou heavy rain. For example, the time series of the number of microblogs on Typhoons Mangkhut [12], Lichma [21], and Mikla [22]all showed a peak in topic discussion one day before the typhoons made landfall. In this event, compared to flooding in coastal areas, there is also a significant lag in the response to storms that fall inland. In this respect, the response time of the flood in Shouguang, Shandong [14]was similar to that of the Zhengzhou rainstorm, but the number of Weibo microblogs during heavy rains in Shenzhen [23] peaked before the heavy rainfall.

This is because the dissemination of disaster warning information by relevant authorities is not timely and occurs through a single channel. According to the survey after the disaster, a number of warning notices only contained the abstract words “pay attention to prevention”, and the warning effect was weak. Second, people were not sensitive to the warning of a 24-h rainstorm in this inland region. Because 24-h rain during the monsoon season is common, the local people lacked a basic understanding of extreme rainstorms. Third, people had little safety awareness and did not take decisive measures based on the red warning, thereby missing the opportunity to avoid a large number of casualties [9].

### 4.2 Topic content analysis

A detailed secondary topic classification demonstrates that when the rainstorm disaster occurred in Zhengzhou, the public not only needed the help of rescue personnel but also the delivery of resources. Compared with previous resource information, in addition to the necessary drinking water, food, and other daily needs information, social media in Zhengzhou also revealed efforts for the collection of menstrual supplies for women. Resources released by official or spontaneously donating merchants and non-profit organisations can also be quickly disseminated through social media. The difference between the type of aid topic and the previous research on the topic of disaster public opinion is reflected in the “Enterprise Donation” type. During the rainstorm in Zhengzhou, more than 70 enterprises spontaneously donated money and material resources, reflecting the increasing support for disaster relief. Analysis of the temporal series of the theme content shows thatextreme rainstorms can cause breakage of the ring in addition to a short period of time, people are also directly affected by it in later stages. Other studies [ [14,15]] have categorized the topic of “Traffic Condition” with less variation in temporal trends during flood formation than in this experiment. In the category “Traffic situation”, the keyword “Zhengzhou Metro” appeared more frequently including the Subway Line 5 disaster event, in which 14 people died, 11 of whom were women. Women have weaker skills in self-help in the event of disasters and higher mortality rates than men. The event has become the focus of public opinion.

### 4.3 Spatial feature analysis

**Fig. 9:**
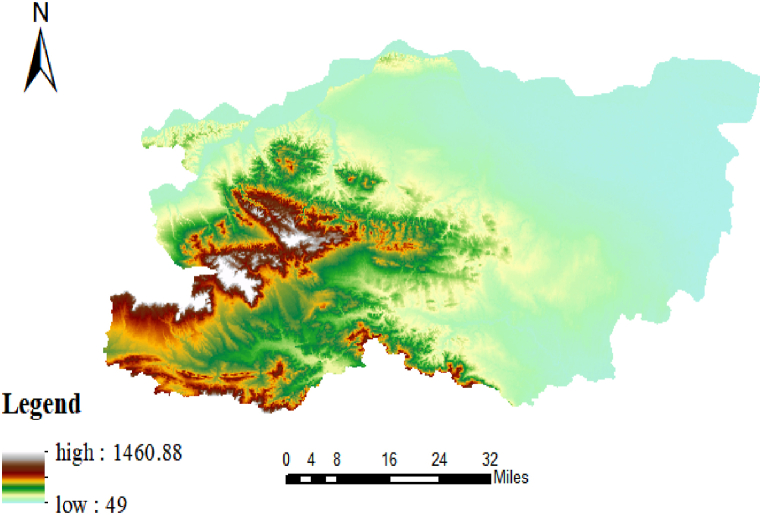
Elevation relief map of Zhengzhou.

Zhengzhou is in the north-central part of Henan Province, with only 38.4% of the plain. However, historically, flooding in China has generally been characterised by more flooding in the east and less in the west, more in the coast and less inland, more in the plains and lakes and less in the highlands and mountains Therefore, Zhengzhou rainstorms may have different formation mechanisms. The results of the post-disaster investigation showed that the heavy rainfall in Zhengzhou City moved from west to east and intensified with the convergence of rivers and floods, combined with the local high topography in the southwest and low topography in the northeast, which belongs to the transition zone from hilly mountains to plains, as shown in Fig. 9, resulting in internal and external flooding [9].

**Fig. 10:**
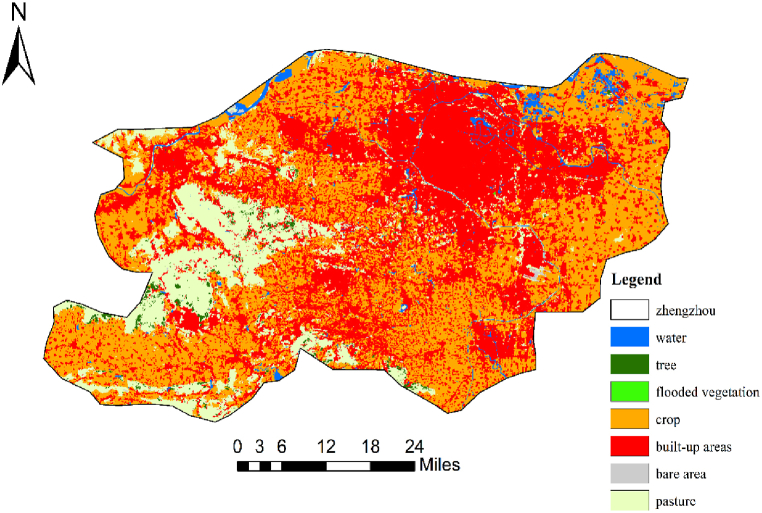
Land use map of Zhengzhou.

In areas with higher levels of urbanisation, dense infrastructure, such as urban buildings and lower vegetation, increases the risk of flooding [24]. According to statistics, from 2000 to 2021, the impervious area of Zhengzhou City will increase from 373 km^2^ to 1147 km^2^, an almost threefold increase [25]. Zhengzhou is an emerging megacity, national central city, international comprehensive transportation hub and logistics centre, and national historical and cultural city whose urbanisation level in 2020 reached about 82%, and the construction of the land is clearly shown in Fig. 10. With the development of the city, the three reservoirs that served Zhengzhou since ancient times have disappeared. It cannot be ignored that the convenient transportation infrastructure and urban network throughout the urbanised area also lack flood discharge channels, which increases the disaster risk during rainstorms.

From the specific spatial distribution of public opinion topics, the rainstorm incident attracted the attention of the people throughout the country, even people not in the affected areas. The people in the disaster areas were more concerned about the needs and distribution of resources and more grateful for actual disaster relief support. The negative conversations about the disaster are concentrated in Beijing, Shanghai and Guangzhou except in the disaster-affected areas, perhaps because Beijing is the capital with more media and news networks and Shanghai and Guangzhou and other coastal cities were prone to be affected by heavy rainfall in the same time Therefore, they understood the situation in inland regions and provided feedback on their concerns regarding this extreme disaster. For example, on the 20th, the Meteorological Observatories of Shenzhen, Haikou, and Changsha issued an early warning signal that upgraded the orange warning of heavy rain in multiple districts to a red warning on the 28th, and Shanghai issued an orange typhoon warning (high wind + heavy rain) on the 24th. These factors resulted in higher kernel density values in all areas.

(The data in Fig. 9 are derived from the Geospatial Data Cloud of the Chinese Academy of Sciences, and the data in Fig. 10 are derived Environmental Systems Research Institute).

### 4.4 Emergency response recommendations

Several emergency responses are suggested by these results and discussion. First, individuals should pay attention to extreme rainstorms. Extreme weather events are occurring with global climate change. Even inland areas have a high risk of extreme rainfall, which is typical in traditional coastal areas. This lesson should be learned by more people so that the public might understand the potential serious consequences caused by extreme meteorological disaster. Therefore, when early warning messages appear, people should understand their meaning under the emergency meteorological conditions. Thus, it is suggested that a more scientific population and emergency disaster risk-reduction education be provided to the public.

Second, disaster prevention and mitigation in urban facilities must be improved. This would include the improvement of the anti-disaster fortification standards for flood prevention, drainage, and public service facilities to achieve the highest degree of urban disaster prevention and mitigation capacity that is compatible with economic and social development; city execution of comprehensive risk surveys of natural disasters to ensure the safe operation of key facilities, such as power supply and drainage pumping stations; strengthening the construction of emergency engineering forces; and improving the disaster response mechanism for important transportation infrastructure, such as subways.

Third, mechanisms for disaster prevention and mitigation in cities should be enhanced. This could be achieved by strengthening the integrated management of early warning and response; enhancing the risk awareness, self-rescue, and mutual rescue capabilities of society as a whole; and carrying out public training on public transportation emergency self-rescue daily. The transparency and efficiency of the distribution mechanisms of donated resources should also be improved.

Fourth, attention should be given to inclusiveness in disaster relief. Early plans should be established for vulnerable groups such as the elderly, women, and children to reduce major casualties during disasters. Some densely populated areas such as high buildings, basements, and transportation tunnels should take special measures to reduce disasters at the community level.

Fifth, multichannel and rapid dissemination of disaster information is necessary through measures that make full use of social media's wide reach and rapid dissemination of news to provide flood warnings and promote disaster prevention, mitigation, and relief knowledge in a variety of ways. Collecting and disseminating information quickly to disaster relief networks will aid the work not only of official rescue personnel, but also of social volunteers by facilitating accurate location-based relief services.

## 5 Conclusion

The impact of extreme rainstorms on cities has increased gradually. Public opinion during the evolution of flood disasters can be obtained to support emergency management decisions. This study takes extreme rainstorms in inland cities in China as the research object, extracting 6 categories of primary topics and 14 categories of secondary topics of flooding disasters using Sina Weibo data and analysing their content and spatial and temporal distribution. The results show that the entire trend of the topic is characterised by periodicity and lags significantly compared to coastal regions. Second, public sentiment gradually shifted from fear to the urgent need for supplies. From content mining, more detailed information can be obtained, such as the requirements of women and news about events like the Subway Line 5 submergence. Third, this extraordinarily heavy rainfall event not only influenced the main affected area, Henan Province, but also received high attention in China's other major urban agglomerations and related disaster-prone coastal areas. Fourth, community-level disaster relief information was also identified, showing that high building power outages, basement floodings, tunnel trapping, and drinking water shortages are common repeated topics in specific inner urban regions. This detailed information will contribute to accurate location-based relief in the future. Finally, based on this lesson, a series of measures for urban flood reduction was proposed, including disaster prevention awareness, infrastructure building, regulatory mechanisms, social inclusivity, and media dissemination. There still has limitation for this study. Owing to the amount of data records in social media is huge, this paper improves the efficiency through iterative experiments. But it is still time-consuming to screen and label the data. In the future, deep learning techniques should be used to improve the topic extraction efficiency.

## Author contribution statement

Zheng Qu; Juanle Wang; Min Zhang: Conceived and designed the experiments; Performed the experiments; Analysed and interpreted the data; Contributed reagents, materials, analysis tools or data; Wrote the paper.

## Data availability statement

Data included in article/supp. Material/referenced in article.

## Additional information

No additional information is available for this paper.

## Funding

This research was funded by the 10.13039/501100001809National Nature Science Foundation of China (No.42050105), and the Construction Project of China Knowledge Centre for Engineering Sciences and Technology (No.CKCEST-2022-1-41).

## Declaration of competing interest

The authors declare that they have no known competing financial interests or personal relationships that could have appeared to influence the work reported in this paper
